# Factors associated with social participation among community-dwelling frail older adults in Japan: a cross-sectional study

**DOI:** 10.1186/s12877-024-04747-2

**Published:** 2024-03-06

**Authors:** Saori Anzai, Hironori Ohsugi, Yoshitaka Shiba

**Affiliations:** 1 Department of Physical Therapy, Faculty of Social Work Studies, Josai International University, 1 Gumyo, Togane, Chiba, 283-8555 Japan; 2 Department of Physical Therapy, Fukushima Medical University School of Health Sciences, 1 Hikariga-oka, Fukushima, 960-1295 Japan

**Keywords:** Aged, Frail older adults, Social activity, Social participation

## Abstract

**Background:**

In recent years, it has become clear that participation in social activities by the older adult suppresses their need for long-term care. Likewise, social participation can promote long-term care prevention among frail older adults who are at a higher risk of needing long-term care. However, their social participation rate is low, and the factors causing these low rates of participation are unclear. Therefore, this study identifies the factors affecting social participation of frail older adults.

**Methods:**

After excluding those certified as requiring long-term care, 28,636 older adults within the target region were selected to receive questionnaires. The questionnaires were distributed and collected via mail. A total of 22,048 respondents (77.0%), including 9,325 men and 10,150 women, were included; 2,655 frail older adults were identified for analysis. Questionnaire items inquired about social participation, basic attributes, need for long-term care, mobility, subjective health, direct and indirect contact with relatives living separately and direct and indirect contact with friends and neighbors. For the statistical analysis, this study employed a binomial logistic regression analysis with social participation as the objective variable.

**Results:**

The rate of social participation among frail older adults was 13.7%. Items related to social participation included sex, economic status, mobility, subjective health, direct contact with friends, and indirect contact with friends.

**Conclusions:**

Interactions with friends and neighbors and physical functionality are correlated with levels of social participation among frail older adults, suggesting that social participation can be promoted by maintaining friendships, forming new ones, and maintaining and improving physical functionality.

## Background

The aging rate in Japan surpassed that of all other countries, reaching 28.4% in 2019. The older population is expected to increase continually and will likely comprise 40% of the population by 2065 [1]. To accommodate the accompanying increase in demand for long-term care, the Japanese government is implementing a long-term care prevention project that seeks to promote social participation among the older adults to lessen the need for long-term care [2].

Considerable research has been conducted on social participation among the alder adults who are not frail, which has been found to promote long-term health, maintenance and improvement of activities of daily living, and quality of life [3–5]. In addition, research into related factors has identified relationship between sex, age, economic status, mobility, health self-assessment, and number of friends [6–9]. In recent years, research on long-term care has found that social participation suppresses the need for long-term care [10]. Although ordinary older adults participate in social activities, the frail older adults, who are at a higher risk of needing long-term care, often have lower rates of social participation [11]. However, the Japanese government has emphasized the need to promote social participation among all older adults, including those who are frail [12]. This emphasis is partly driven by studies, such as one that reported how social participation can prevent functional disability among frail older adults [13]. Therefore, promoting social participation among this population has become a crucial issue.

Most studies on social participation among the older adults have focused on the older adults in general, with few studies specifically focusing on frail individuals [13–15]. One study [14] explored this gap by interviewing frail older adults and highlighting the influence of physical environments, such as traffic and local stores, and social networks, on their social participation. Another study, focusing on support-needing frail adults, delved deeper, revealing the complex interplay between care needs, social interactions, and daily living functions within the certification categories [15]. However, no research has been conducted on the frail older adults, considering the stage prior to being in need of support/long-term care, and the factors in their social participation have not been clarified. In the future, it will be essential to clarify the factors related to social participation of frail older adults to promote long-term care prevention through social participation.

Therefore, this study sought to examine the factors affecting social participation among frail older adults.

## Methods

### Study design

A cross-sectional study.

### Research participants and survey method

A total of 28,636 residents of City B, Prefecture A, aged 65 or older participated in the survey. All participants were not receiving certified long-term care, regardless of their use of long-term care services. Participants included both those aged 65 or older without certified care needs and those officially recognized as requiring support. In 2018, questionnaires were sent to potential research participants and collected via mail. Among the 22,048 respondents (77.0% response rate), 9,325 were males and 10,150 were females. Of these, there were 19,385 valid responses for the social participation and frailty screening index [12], from which 2,655 frail older adults were selected for analysis.

The survey participants were informed in writing of the study’s purpose and that their personal information would be protected. Respondents’ agreement to cooperate was confirmed as part of the process of filling out the questionnaire. informed consent was obtained.

This study was approved by the Josai International University Board of Ethics (No. 2022-004).

### Measurement items

#### Social participation

Using the items of the “care prevention/daily living area needs survey” conducted in Japan to measure the health status of the older adults [16], four types of social participation (volunteer groups, sports-related groups or clubs, hobby-related groups, and learning/study-related clubs) were measured using a six-point scale (1. Four or more times a week, 2. Two or three times per week, 3. Once a week, 4. One to three times per month, 5. A few times a year, and 6. Not at all), with participation in an activity once a month or more was considered “participation” and participation that was less than once a month was considered “no participation.” Participation in one or more types of activities decreases the risk of incident functional disability [17], respondents were identified as engaged in social participation if they participated in at least one of the four types of activities.

#### Frailty

Frailty was investigated using the frailty screening index [18], developed based on the Cardiovascular Health Study criteria [19], the Kihon checklist [20], and other Japanese questionnaires. The index consisted of five items: (1) “Have you lost 2 kg or more in the past six months?”, (2) “Do you think you walk slower than before?”, (3) “Do you go for a walk for your health at least once a week?”, (4) “Can you recall what happened five minutes ago?”, and (5) “In the past two weeks, have you felt tired without a reason?”.

Scores of three or more were defined as frail, 1 to 2 as prefrail, and 0 as robust, per the Cardiovascular Health Study criteria [19]. This study analyzed frail older adults, defined as those who scored three or more.

#### Independent variables

The following items, thought to be related to social participation, were investigated as independent variables with reference to previous research.

The basic attributes investigated were sex, age, household composition, and subjective economic status. Household composition was divided into “living alone” and “living with others.” The question on economic status asked respondents was as follows: “How do you feel about your current lifestyle in economic terms?” The respondents answered on a five-point scale (1. Very difficult; 2. Somewhat difficult; 3. Ordinary; 4. Somewhat well off; 5. Very well off). Answers 1 and 2 were categorized as “economic difficulty,” and Answers 3–5 were categorized as “no economic difficulty.” The need for long-term care and mobility was investigated to further explore the effects of physical fitness on long-term care. Self-reported responses to the question “Do you need care or assistance in daily life?” were categorized into “no need for care” and “requiring care.” Mobility was investigated using the physical function of the Kihon Checklist [20]. A respondent’s mobility was categorized as “declining” if three or more items were checked, and “no decline” when three or more items were not checked [21]. Subjective health was investigated according to psychological aspects. Respondents answered the question “How is your health at this point?” on a four-point scale (1. Very good, 2. Good enough, 3. Not very good, and 4. Bad). Answers 1 and 2 were categorized as “in good health,” while Answers 3 and 4 were categorized as “in poor health.” Social networks were used to investigate the social aspects. Direct and indirect contact with relatives who live elsewhere, as well as with friends and neighbors, was investigated. With regards to both direct and indirect contact, respondents were asked, respectively, “How often do you have the chance to meet in person or go out together?” and “How often do you have the chance to get in touch by letter, telephone, e-mail, etc.?” These questions were answered using a seven-point scale (1. Almost every day, 2. Two or three times a week, 3. Approximately once a week, 4. Two or three times per month, 5. Approximately once a month, 6. A few times a year, and 7. Not at all). Responses were subsequently categorized as either “once per week or more” or “less than once per week.”

### Data analysis methods

The relationship between participants’ characteristics and their degree of social participation was analyzed using t-tests or chi-square tests. To identify the factors relevant to social participation, binomial logistic regression analysis (forced entry method) was conducted with social participation as the objective variable. Since mobility and the need for care were highly related, the need for care was not entered into the logistic regression analysis. SPSS Statistics 26.0 for Windows was used for all statistical analyses, with a significance level set at < 5%.

## Results

### Status and nature of social participation among the frail older adults

Table 1 presents a comparison of the basic attributes and independent variables related to social participation. Of the 2,655 frail older adults, 545 (20.5%) participated in this study. Levels of social participation varied significantly for the following questionnaire items: sex, economic status, need for long-term care, mobility, subjective health, indirect contact with relatives living separately, direct contact with friends and neighbors, and indirect contact with friends and neighbors. No significant differences were found according to age, household composition, or direct contact with relatives living separately.

**Table 1:** Status and nature of social participation in frail older adults

	Total (*n* = 2,655)			Participation (*n* = 545)			Nonparticipation (*n* = 2,110)			*P*-value	Effect size	95%CI
Age(mean ± SD)^*^	75.8	±	7.0	76.0	±	6.5	75.8	±	7.1	0.637*	0.02†	-0.61–0.64
Sex, *n*(%)												
Men	1211		(45.6)	213		(39.1)	998		(47.3)	0.001	0.07	N/A
Women	1444		(45.6)	332		(60.9)	1112		(52.7)			
Household composition, *n*(%)												
Living with others	2067		(78.6)	422		(77.6)	1645		(78.8)	0.519	0.01	N/A
Living alone	564		(21.4)	122		(22.4)	442		(21.2)			
Economic states, *n*(%)												
Economic difficulty	995		(38.0)	145		(27.1)	850		(40.8)	< 0.001	0.11	N/A
No economic difficulty	1623		(62.0)	390		(72.9)	1233		(59.2)			
Need for long-term care, *n*(%)												
Requiring care	861		(33.4)	145		(27.5)	716		(35.0)	0.001	0.06	N/A
No need for care	1714		(66.6)	382		(72.5)	1332		(65.0)			
Mobility, *n*(%)												
Decline	587		(22.1)	82		(15.0)	505		(23.9)	< 0.001	0.09	N/A
Not decline	2068		(77.9)	463		(85.0)	1605		(76.1)			
Subjective health, *n*(%)												
Poor	1264		(48.9)	189		(36.1)	1075		(52.2)	< 0.001	0.13	N/A
Good	1320		(51.1)	334		(63.9)	986		(47.8)			
Direct contact with relatives living separately, *n*(%)												
Less than once a week	1923		(76.3)	384		(73.8)	1539		(77.0)	0.148	0.03	N/A
More than once a week	597		(23.7)	136		(26.2)	461		(23.1)			
Indirect contact with relatives living separately, *n*(%)												
Less than once a week	1758		(69.8)	323		(62.4)	1435		(71.7)	< 0.001	0.08	N/A
More than once a week	762		(30.2)	195		(37.6)	567		(28.3)			
Direct contact with friends and neighbors, *n*(%)												
Less than once a week	1908		(74.0)	262		(49.1)	1646		(80.5)	< 0.001	0.29	N/A
More than once a week	670		(26.0)	272		(50.9)	398		(19.5)			
Indirect contact with friends and neighbors, *n*(%)												
Less than once a week	1941		(75.7)	310		(58.6)	1631		(80.1)	< 0.001	0.20	N/A
More than once a week	624		(24.3)	219		(41.4)	405		(19.9)			

* *P*-value is based on Independent T-test

*P*-values are based on χ^2^ test

† Effect size is based on *r* value

Effect size is based on φ

N/A: Not Applicable

### Factors relevant to social participation among the frail older adults

Table 2 shows the results of the logistic regression analysis. Adjusted for all independent variables, the odds ratios were 1.41 (95% Cl 1.11–1.79) for economic status, 1.41 (95% Cl 1.03–1.922) for mobility, 1.49 (95% Cl 1.19–1.88) for subjective health, 3.15 (95% Cl 2.43–4.09) for direct contact with friends and neighbors, and 1.41 (95% Cl 1.07–1.86) for indirect contact with friends and neighbors. Table 3 shows the results of the logistic regression analysis for each type of social activity. Significant associations with functional decline varied depending on the type of social activity.

**Table 2:** Logistic regression analysis for factors relevant to social participation among the frail older adults

	OR	95%CI	*P*-value	VIF
Age	1.01	0.99–1.03	0.172	1.141
sex				
men	1.00			
women	1.25	0.84–1.41	0.055	1.070
household composition				
Living with others	1.00			
Living alone	1.09	0.84–1.41	0.529	1.025
economic states				
economic difficulty	1.00			
no economic difficulty	1.41	1.11–1.79	0.005	1.094
Mobility				
Decline	1.00			
not decline	1.41	1.03–1.92	0.031	1.206
subjective health				
Poor	1.00			
Good	1.49	1.19–1.88	0.001	1.155
direct contact with relatives living separately				
less than 1 a week	1.00			
more than 1 a week	0.82	0.61–1.11	0.199	1.426
indirect contact with relatives living separately				
less than 1 a week	1.00			
more than 1 a week	0.99	0.75–1.31	0.938	1.511
direct contact with friends and neighbors				
less than 1 a week	1.00			
more than 1 a week	3.15	2.43–4.09	< 0.001	1.416
indirect contact with friends and neighbors				
less than 1 a week	1.00			
more than 1 a week	1.41	1.07–1.86	0.014	1.459

χ^2^ = 207.98 (p < 0.001)

The dependent variable was participation in activities. Adjusted for all independent variables

OR, Odds ratio, 95% CI, 95% Confidence interval

**Table 3:** Logistic regression analysis for each type of social activity among the frail elderly

	volunteer groups			sports-related groups or clubs			hobby-related groups				learning/study-related clubs		
	OR	95%CI	*P*-value	OR	95%CI	*P*-value	OR	95%CI	*P*-value		OR	95%CI	*P*-value
age	1.03	1.00–1.06	0.086	0.99	0.96–1.01	0.280	1.02	1.00–1.04	0.102		0.99	0.97–1.02	0.586
sex													
men	1			1			1				1		
women	0.96	0.64–1.45	0.859	0.92	0.65–1.30	0.628	1.54	1.18–2.02	0.002		1.95	1.32–2.88	0.001
household composition													
Living with others	1			1			1				1		
Living alone	1.04	0.65–1.68	0.858	0.97	0.64–1.47	0.884	1.04	0.76–1.42	0.799		1.12	0.72–1.72	0.619
economic states													
economic difficulty	1			1			1				1		
no economic difficulty	1.01	0.65–1.56	0.970	2.01	1.34–3.01	0.001	1.58	1.18–2.11	0.002		1.94	1.25–3.01	0.003
mobility													
decline	1			1			1				1		
not decline	2.80	1.35–5.81	0.005	1.26	0.77–2.09	0.360	1.40	0.96–2.03	0.078		1.98	1.09–3.63	0.026
subjective health													
poor	1			1			1				1		
good	1.26	0.83–1.93	0.280	1.13	0.79–1.62	0.494	1.48	1.13–1.95	0.005		1.29	0.87–1.90	0.202
direct contact with relatives living separately													
less than 1 a week	1			1			1				1		
more than 1 a week	0.90	0.53–1.55	0.716	1.09	0.70–1.72	0.693	0.74	0.52–1.05	0.088		0.89	0.54–1.45	0.629
indirect contact with relatives living separately													
less than 1 a week	1			1			1				1		
more than 1 a week	0.94	0.57–1.56	0.812	0.88	0.57–1.35	0.553	0.93	0.67–1.28	0.643		0.81	0.51–1.27	0.355
direct contact with friends and neighbors													
less than 1 a week	1			1			1				1		
more than 1 a week	3.02	1.87–4.86	< 0.001	3.79	2.54–5.67	< 0.001	3.22	2.39–4.36	< 0.001		2.53	1.65–3.90	< 0.001
indirect contact with friends and neighbors													
less than 1 a week													
more than 1 a week	1.30	0.80–2.13	0.293	0.94	0.62–1.43	0.773	1.58	1.16–2.17	0.004		1.65	1.06–2.56	0.026

OR, Odds ratio, 95% CI, 95% Confidence interval

The dependent variable was participation for each type activity. Adjusted for all independent variables

## Discussion

In this study, 20.5% of frail older adults participated in social activities. A survey by the Japanese Cabinet Office reported a social participation rate of 58.3% among older adults over 60 [22]. Although this study targeted a different age group, it is clear that only a small minority of the frail older adults targeted in this study are participating in society. Tomioka et al. reported that the older adults with a higher capacity for instrumental activities of daily living (IADL) are more likely to participate in society [7]. Frailty is thought to reduce IADL capacity, thus making social participation difficult. Approximately 30% of the study participants were in need of some kind of long-term care, suggesting decreased IADL capacity. In the present study, it was estimated that this influenced the low rate of social participation among frail older adults.

In recent years, the Japanese public policy has been oriented toward preventing the need for long-term care by promoting the participation of frail older adults in social activities [12]. This study confirms the low participation rate of frail older adults in social activities. Identifying relevant factors promoting their social engagement is crucial. This study used logistic regression analysis to examine the factors related to social participation in frail older adults. Consequently, the following relevant factors were identified: mobility, subjective health, and direct or indirect contact with friends. This article first addresses basic attributes.

For basic attributes, a significant relationship was observed between economic status and social participation. This is further reflected in the analysis by type of social participation, which revealed significant associations among sports-related groups or clubs, hobby-related groups, and learning/study-related clubs. It is logical to infer that many such activities involve costs, making economic comfort a prerequisite for engagement.

Interestingly, the results of this study align with previous research [8] by highlighting economic status as a risk factor for functional disability. However, unlike many other influencing factors, economic status at an older age is often difficult to significantly improve. his could explain why, in our findings, the odds ratio for economic status (1.41) remained lower compared to other factors. This difference suggests that the disadvantages of economic status can be partially compensated for by actively cultivating other modifiable factors, such as maintaining strong social connections through regular contact with friends.

Next, we discuss health-related aspects of mobility and subjective health. Furthermore, the nature of social interaction appeared to play a role. Previous research has identified a relationship between social participation, mobility, and subjective health in univariate analysis, but not in multivariate analysis [8]. Therefore, the significant interrelation between these factors may be a particular characteristic of frail older adults. However, in previous study targeting frail older adults [14], no relationship between subjective health and social participation has been reported. It is unclear whether this is a characteristic of a particular study subjects or the target region.

Turning to the social aspect of direct and indirect contact with friends, this study investigated participants’ interactions with relatives living separately, and with friends and neighborhood acquaintances as social network variables, identifying the latter as a relevant factor. Direct contact was significantly associated with all types of social participation, while indirect contact had significant associations with hobby-related groups, and learning/study-related clubs. Previous research targeting the urban older adults has reported [9] that the number of close friends a person has is related to their social participation levels, which supports the results of this study. Watanabe et al. [23] have shown that “suggestions made by acquaintances” can lead to participation in regional activities. Therefore, the stronger one’s friend network, the easier it is to be invited to participate in social activities. Accordingly, maintaining a friend network and making new friends can significantly promote social participation among frail older adults. However, frail older adults have fewer opportunities to leave the house compared to the older adults in general [24]. Consequently, their friend networks tend to weaken. In addition, they have difficulty finding opportunities to make new friends; therefore, maintaining and improving their friend networks is difficult. Rather than relying solely on invitations from friends, interventions by third parties such as administrative agencies are essential. Japan’s past high-risk approach to long-term care prevention involved identifying frail older adults through surveys and directly encouraging them to participate in activities. In the future, regional activities for local residents, now expected to become the locus of long-term care prevention, will need to promote participation through third parties such as administrative agencies to involve the frail older adults. However, our measure of direct contact with friends in this study (“How often do you have the chance to meet in person or go out together?”) likely captured some interactions that occurred within the context of social activities. Many respondents likely met friends through those activities. This overlap raises an interesting point: while one previous study [14] suggests that “the cause of diminishing activity was the decline of social networks,” our findings further support the idea that maintaining these networks, including direct contact with friends, plays a crucial role in promoting social participation.

Moreover, this study found that frequent indirect contact with friends and neighbors was also related to social participation. Maintaining a friend network, even though indirect interaction, makes it easier to receive “invitations from acquaintances,” leading to increased social participation. The frail older adult is less able to leave their houses [24] and have fewer opportunities than other older adults to meet friends in person. However, this study demonstrates that even indirect contact can cause higher rates of social participation among frail older adults. In this study, telephone, e-mail was used as examples of indirect contact, and cell phones and smartphones are often used for these methods of communication. In this study, telephone and e-mail were used as examples of indirect contact, with cell phones and smartphones often serving as tools for these communications. However, a previous study showed that while 80% of older adults use smartphones, usage of specific services like e-mail and messenger varies greatly, suggesting individual differences in proficiency [25]. This means that while those with high proficiency could leverage smartphones for indirect interaction, those with low proficiency may have struggled, potentially affecting our study results. Despite this potential limitation, research [26] has demonstrated the potential of digital devices like smartphones to reduce social isolation in older adults. Therefore, promoting indirect contact through accessible digital tools could be an important strategy for encouraging social participation among frail older adults. However, this cross-sectional study could not measure the causes and effects of friends’ networks and social activities. Further research should be conducted longitudinally to clarify these relationships.

In addition, logistic regression analysis revealed no relationship between interactions with relatives living separately and level of social participation. Networks with relatives living separately are a compositional element of social isolation, which is one of the risk factors for needing long-term care. Therefore, it is important from the perspective of long-term care prevention. In addition, previous research has reported that the presence or absence of a spouse is related to participation in social activities [27]; the presence of close family members may encourage social participation. However, no relationship was found in this study, suggesting that for frail older adults, enhancing communication with relatives will not lead to increased social participation.

This research clarified the characteristics of social participation among frail older adults to identify possible approaches for promoting participation in social activities among this group. The results suggest that social activities are related to interactions with friends and neighbors and physical functionality. Therefore, maintaining friends’ networks, making new friends, and maintaining and improving physical functionality could promote participation in social activities.

The limitations of this study are as follows: As a cross-sectional study, this study, the causes and effects of the factors found to be related to social activities could not be confirmed. Although close friend networks are thought to lead to increased participation in social activities, it also seems that participation in regional social activities contributes to the enhancement of friend networks.

The second limitation of this study concerns the self-administered survey method. As the target population consisted of frail older adults, the high association between cognitive function and frailty noted in a previous study [28], suggests that some participants with cognitive decline may have been included. This could have potentially affected the accuracy of their responses. The third limitation concerns the selection of independent variables. The factors related to social participation identified in this study were similar to those identified in the research on non-frail older adults. However, there are significantly fewer frail older adults currently participating in social activities compared to their non-frail counterparts. Correspondently, there may be underlying factors related to their social participation which were not covered in this survey. Therefore, there are likely factors related only to social participation of the frail older adults that have not been identified in surveys of the older adults overall. To clarify this point, qualitative investigations, such as interviews with frail older adults, are required to identify these new related factors. Once identified, these factors should be investigated quantitatively.

## Conclusion

Factors associated with social participation among the frail older adults included gender, economic status, mobility, subjective health, direct contact with friends, and indirect contact with friends. The results suggest that maintaining a network of friends, making new friends, and maintaining or improving physical function may promote the social participation among frail older adults.

However, there may be underlying factors related to social participation among the frail older adults that were not discussed in this study. To clarify this point, qualitative studies such as interviews with frail older adults should be conducted to identify new relevant factors.

## Data Availability

The data that support the findings of this study are available from B city, A Prefecture, Japan but restrictions apply to the availability of these data, which were used under license for the current study, and so are not publicly available. Data are however available from the authors upon reasonable request and with permission of B city, A Prefecture, Japan through a request to anzai@jiu.ac.jp [Saori Anzai].

## Abbreviations

IADL: Instrumental activities of daily living
